# Supplementary material for: Chromothripsis during telomere crisis is independent of NHEJ, and consistent with a replicative origin
Source: Genome Res. 2019 May;29(5):737–49. doi: 10.1101/gr.240705.118 (PMC6499312; doi:10.1101/gr.240705.118)
Supplement: Supplemental Material [file supp_gr.240705.118_Supplemental_file_1.zip › contigs/annotated_contigs/DB114/contig.2.DB114_length_436_mean_cov_5.46100917431.docx]

**DB114_length_436_mean_cov_5.46100917431**

```
AAAGAAAAAGAAAAAGAAAAAGAAAAAGAAAACCAAAGGGACTCGTCCGCAGCGTTATCTGCTGGCGCCATCTTCCGGAAATAAGTTAG
 >chr20:43496323-43496589 + E=1e-148
CACAACAGGTCTATTTCTGTTTCAGCGGGGCGTGAGGGCACTGGAAATGCAGGCAAGGAGGGGAAAAGAGAGAATGAATTTTAGCAAAA

TCTCTCCATCTCCTCTGTCCTGGATCATCTAATTACTCTACCAAGATTATCATTTTAATGTAATAGTACTACTAACATGCTAGCA|CTT
                                                                                      >c
|TGACATTCTCAAATAAGAAATGGAAGAATTGCCAAGGCACAGTGGCTCACGCCTGTAATCCTAACCCTTTGGGAGTCCAAGGCGGGGG
hr20:43497578-43497744 + E=4e-63
GATCACTTGAGGTCAGGCGTTTGAGACCAGCCTGGCCAACATGATGAAACCCCCATCTCTACTAAAAACACAAAAA|TTAGGC
```
